# Access and Barriers to Use of Long Term Services: Contextual Issues

**DOI:** 10.1093/geroni/igab046.1655

**Published:** 2021-12-17

**Authors:** Allen Glicksman, Lauren Ring, Norah Keating

## Abstract

The challenges that some older adults face in accessing both health and social services is a topic of continuing concern. This panel will focus on contextual issues that often shape specific challenges. These contextual issues usually emerge either from issues of diversity among the older persons themselves (for example, minority status or foreign born) and diversity between the ways in which services are offered (usually established at the national or in the case of the United States, at the state level). The intersection of these two forms of diversity often define the specific challenges faced by older persons in accessing health and social services. Further, unexpected events, such as the COVID pandemic, can affect both types of diversity (greater challenges for persons who do not speak the dominant language; inability of services to quickly adapt to radically changed environment). Our panel will address these issues through four presentations, each taking a different look at the ways in which diversity affects access. Our first paper, by Torres, will place this discussion in wider context by presenting results from a scoping review. Our second paper, by Diederich looks at access to services by immigrant generation (that being another source of diversity) in Germany. The third paper, by Thiamwong looks at how the COVID crisis affected older Hispanic women. Finally, Ring will examine how a national policy, here the definition of poverty, affects outcome and access for older person in the United States.

